# The spatial signature of *Plasmodium vivax* and *Plasmodium falciparum* infections: quantifying the clustering of infections in cross-sectional surveys and cohort studies

**DOI:** 10.1186/s12936-023-04515-4

**Published:** 2023-03-04

**Authors:** Mirco Sandfort, Wuelton Monteiro, Marcus Lacerda, Wang Nguitragool, Jetsumon Sattabongkot, Andreea Waltmann, Henrik Salje, Amélie Vantaux, Benoit Witkowski, Leanne J. Robinson, Ivo Mueller, Michael White

**Affiliations:** 1 Unité Malaria : Parasites Et Hôtes, Département Parasites Et Insectes Vecteurs, Institut Pasteur, Paris, France; 2 Sorbonne Université, Collège Doctoral, Paris, France; 3 Fundação de Medicina Tropical Dr. Heitor Vieira Dourado, Manaus, Brazil; 4 Universidade do Estado do Amazonas, Manaus, Brazil; 5 Instituto de Pesquisas Leônidas e Maria Deane, Manaus, Brazil; 6 Department of Molecular Tropical Medicine & Genetics, Faculty of Tropical Medicine, Mahidol University, Bangkok, Thailand; 7 Mahidol Vivax Research Unit, Faculty of Tropical Medicine, Mahidol University, Bangkok, Thailand; 8 Population Health & Immunity Division, Walter and Eliza Hall Institute of Medical Research, Parkville, Australia; 9 Department of Medical Biology, University of Melbourne, Melbourne, Australia; 10 Department of Genetics, University of Cambridge, Cambridge, UK; 11 Malaria Molecular Epidemiology Unit, Institut Pasteur du Cambodge, Phnom Penh, Cambodia; 12 Burnet Institute, Melbourne, Australia; 13 G5 Épidémiologie et Analyse des Maladies Infectieuses, Département de Santé Globale, Institut Pasteur, Paris, France

**Keywords:** Malaria, *Plasmodium vivax*, *Plasmodium falciparum*, Spatial epidemiology, Spatial clustering, Spatiotemporal clustering, Spatial signature

## Abstract

**Background:**

Over the last decades, enormous successes have been achieved in reducing malaria burden globally. In Latin America, South East Asia, and the Western Pacific, many countries now pursue the goal of malaria elimination by 2030. It is widely acknowledged that *Plasmodium* spp. infections cluster spatially so that interventions need to be spatially informed, e.g. spatially targeted reactive case detection strategies. Here, the spatial signature method is introduced as a tool to quantify the distance around an index infection within which other infections significantly cluster.

**Methods:**

Data were considered from cross-sectional surveys from Brazil, Thailand, Cambodia, and Solomon Islands, conducted between 2012 and 2018. Household locations were recorded by GPS and finger-prick blood samples from participants were tested for *Plasmodium* infection by PCR. Cohort studies from Brazil and Thailand with monthly sampling over a year from 2013 until 2014 were also included. The prevalence of PCR-confirmed infections was calculated at increasing distance around index infections (and growing time intervals in the cohort studies). Statistical significance was defined as prevalence outside of a 95%-quantile interval of a bootstrap null distribution after random re-allocation of locations of infections.

**Results:**

Prevalence of *Plasmodium vivax* and *Plasmodium falciparum* infections was elevated in close proximity around index infections and decreased with distance in most study sites, e.g. from 21.3% at 0 km to the global study prevalence of 6.4% for *P. vivax* in the Cambodian survey. In the cohort studies, the clustering decreased with longer time windows. The distance from index infections to a 50% reduction of prevalence ranged from 25 m to 3175 m, tending to shorter distances at lower global study prevalence.

**Conclusions:**

The spatial signatures of *P. vivax* and *P. falciparum* infections demonstrate spatial clustering across a diverse set of study sites, quantifying the distance within which the clustering occurs. The method offers a novel tool in malaria epidemiology, potentially informing reactive intervention strategies regarding radius choices of operations around detected infections and thus strengthening malaria elimination endeavours.

**Supplementary Information:**

The online version contains supplementary material available at 10.1186/s12936-023-04515-4.

## Background

Globally, enormous successes have been achieved over the last decades in reducing the malaria burden [1]. The main instruments were the distribution of long-lasting insecticidal nets and clinical case management after passive case detection. The strategies deployed so far have proven particularly successful in battling *Plasmodium falciparum* transmission. However, *Plasmodium vivax* causes relapses [2] and to a higher degree asymptomatic low-parasitaemia infections that escape passive case detection but contribute to transmission [3, 4]. The effectiveness against *P. vivax* malaria has thus been less pronounced so that *P. vivax* has predominance over *P. falciparum* in many areas [1].

In Latin America, South East Asia, and the Western Pacific, the overall reduction of malaria burden in many countries has paved the way for the ambitious goal of malaria elimination by 2030 [1, 5]. The necessity to incorporate active finding of infections in order to tackle the silent reservoir of on-going transmission is increasingly acknowledged [3, 4, 6–9]. One strategy considered as part of current recommendations for malaria elimination are reactive interventions, i.e. active finding, testing, and treatment of other infections in a delimited area around passively detected malaria cases [10]. However, the radius of the area in which to optimally implement interventions remains unclear.

Infection with human *Plasmodium* spp*.* through an *Anopheles* mosquito bite depends on a complex interplay of factors of the pathogen, host, vector, and environment. Each of these factors, such as proximity to the vector’s breeding grounds, bed net usage, *Anopheles* species composition, and host immunity, can vary geographically [11, 12]. Geographical heterogeneity in malaria infections appears across multiple spatial scales varying from between households [13–16] to within and between villages [9, 11]. At low incidence, the possibility to identify single malaria transmission clusters has long been reported [17].

Reducing malaria transmission country-wide usually leads to a geographical fragmentation of areas where malaria risk remains high, further increasing spatial heterogeneity [18]. Tailoring interventions to high-risk areas within these countries is at the core of recommended strategies for malaria elimination [10, 19]. Obtaining a good understanding of the spatial dependence between malaria infections will help underpin spatially targeted control efforts.

To date, most efforts to characterise the spatial clustering of malaria have relied on comparing infection prevalence at community or household scales [11, 13]. In addition, some studies have used space–time clustering statistics such as the *k*-nearest neighbour method for binary testing whether spatiotemporal clustering of cases occurred [17]. The introduction of spatial scanning [20, 21] to malaria epidemiology further stimulated studies on spatial heterogeneity in disease or infection risk. This method scans the study area for (size-variable) circular areas with statistically significant clustering of infections (‘hotspots’). Such hotspots were identified in diverse study areas in South America [22–26], Africa [27–39], and South East Asia [9, 40–44], for *P. falciparum* [25, 32, 40, 41], *P. vivax* [25, 26, 32, 40, 41], and *Plasmodium malariae* [40, 41]. However, the effectiveness of the method in forecasting hotspots remains unclear and it does not generalize spatial patterns between infections.

In this study, a global clustering statistic is introduced to malaria epidemiology, borrowing strongly from earlier development in the epidemiology of dengue and other pathogens [45, 46]. The spatial signature quantifies both the magnitude of clustering of infections and the radius within which it is statistically significant, potentially informing reactive interventions as part of malaria elimination endeavours. Spatial signatures of PCR-confirmed *P. vivax* and *P. falciparum* infections were revealed by assessing the pooled prevalence of infections around confirmed index infections in widening spatial and temporal windows. Spatial and temporal clustering of infections was demonstrated in both cross-sectional surveys and cohort studies from a diverse spectrum of study sites in Brazil [6, 47], Thailand [7, 8], Cambodia [9], and Solomon Islands [48].

## Methods

### Cross-sectional malaria prevalence surveys

Data from 5 cross-sectional surveys from 4 countries, i.e. Brazil, Thailand, Cambodia, and Solomon Islands were used. The study design and basic descriptions are summarized in Table 1. A cross-sectional survey in 17 villages in the high-incidence, rural province Mondulkiri, in North-Eastern Cambodia, in the dry season in December 2017 until April 2018 was included (as described in [9]). The 4200 study participants were 2–79 years old and from randomly sampled households based on a census prior to the survey. Villages of residence were classified into categories of proximity to the forest based on a forest cover remote sensing analysis [49]. In this and all following included studies, finger-prick blood samples were tested for *P. vivax* and *P. falciparum* infections by real-time PCR and GPS locations of households were collected.

**Table 1:** Design and basic summary of included cross-sectional surveys and cohort studies

Study type	Country (reference)	Number of participants	Timing and duration	*P. vivax* PCR prevalence (%)	*P. falciparum* PCR prevalence (%)	Median age [range]	Gender [%]
Cross-sectional surveys (sampling once)	Cambodia (9)	4200	Dec 2017–Apr 2018 (dry season)	6.4	3.0	22 years [2–79]	Female 53
	Thailand (7)	4309	Sep–Oct 2012 (low transmission season)	3.1	0.9	20 years [0–92]	Female 52
	Brazil (6)	2010 (Survey 1)	Nov 2012–Jan 2013 (rainy season)	4.3	0.8	23 years [0–100†]	Female 47
		2073 (Survey 2)	Aug–Sep 2013 (dry season)	3.4	< 0.1	25 years [0–100†]	Female 48
	Solomon Islands (48)	3501	May–June 2012 (minimal seasonality)	13.4	0.1	18 years [1–100]	Female 53
Cohort studies (monthly sampling)	Thailand (8)	999 (14 sampling visits)	May 2013–Jun 2014	1.7–4.2*	0–1.3*	23 years [1–82]	Female 54
	Brazil (47)	1274 (13 sampling visits)	Apr 2013–Mar 2014	2.5–6.5*	0–1.0*	25 years [0–100†]	Female 49

^*^Range of monthly prevalence

^†^Some individuals had reported ages greater than 100 years. In these cases, record keeping was considered not sufficiently robust to provide an accurate estimate of age

From Kanchanaburi and Ratchaburi provinces in western Thailand, data from a cross-sectional survey in September–October 2012 were considered [7]. In this survey, 4309 study participants aged 0–92 years were enrolled.

Data were added from two cross-sectional surveys in a peri-urban part of Manaus, Amazonas State, Brazil from November 2012 until January 2013 (rainy season) and from August until September 2013 (dry season), respectively [6]. After all inhabitants had been invited to the study, 2010 and 2073 participated in the two surveys, respectively (963 in both surveys).

From Solomon Islands, data were used from a cross-sectional survey in several villages across Ngella, Central Islands province from May until June 2012 [48]. From a representative household-based sample, 3501 individuals agreed to participate.

### Longitudinal cohort studies

In addition to the cross-sectional surveys, data were used from two longitudinal cohort studies with similar design: Monthly active follow-up with finger-prick blood-sampling for *P. vivax* and *P. falciparum* real-time PCR testing and collection of household locations by GPS at baseline. One study was conducted in two villages in Kanchanaburi and Ratchaburi provinces, Thailand, respectively, in May 2013 until June 2014, enrolling 999 participants (Table 1) [8]. The other study took place in the peri-urban part of Manaus, Brazil, from April 2013 until March 2014 with 1274 enrolled participants [47].

### Spatial signature of malaria infection

In order to characterise the spatial dependence between infections in both the cross-sectional surveys and for each follow-up period in the longitudinal studies, the average prevalence $\pi (d)$ of PCR-positive infections was calculated within a distance $d$ of PCR-positive “index” infections. To do this, the study participants that lived within distance $d$ of each PCR-positive participant were identified, and the proportion that were also PCR-positive was calculated. $d$ was varied in incremental steps by 1 km for $\pi$ across the entire study region; and by 50 m for $\pi$ within 1 km.

The observed prevalence was compared with that expected under a null distribution where no spatial dependence exists. A bootstrap null distribution of $\pi$ was generated after 10,000 random re-allocations of infection locations and statistical significance was defined outside of the null’s 95th percentile interval. 

For the cohort studies, the spatial signature for *P. vivax* infections was calculated as above, pooling across the monthly study visits, i.e. considering only index-neighbour pairs in the same study month. If multiple monthly PCR results were positive for an individual, only the first, “incident” positive result was considered. 

The underlying distribution of pairwise distances between the study participants per study site as well as the underlying distribution of pairwise distances between infections and all study participants are provided in the Additional file 1: Figures S1.1a-S1.7.

### Spatiotemporal signature of malaria infection

In the two longitudinal cohort studies, the extent of spatiotemporal dependence was also explored. The period prevalence $\rho (d, t)$ of PCR-positive neighbouring infections was calculated within a distance $d$ and within a time window $t$ of all incident PCR-positive index infections for *P. vivax* as the ratio of the number of pairs of index and neighbouring incident infections within $d$ and $t$ and the number of pairs of study participants within $d$ (steps by 1 km for $\rho$ across the entire study region; by 50 m for $\rho$ within 1 km). A study participant was considered an incident infection at the first study visit with a PCR-positive blood sample. A bootstrap null distribution of $\rho$ was generated after 1000 random re-allocations of locations of infection time series.

From the cohort study in Thailand, PCR-positive blood samples for *P. vivax* were genotyped as described in [8]. Spatiotemporal signatures were calculated as described above but respecting only *P. vivax* infection pairs with a matching genotype.

### Comparative analysis of distance to 50% reduction in prevalence

From each spatial signature across the cross-sectional surveys and cohort studies, the distance was drawn within which the prevalence around index infection has fallen by 50% (between the prevalence at 0 m and the study’s global prevalence). The distance was approximated as the mean of the pair of 50 m-increments of distance $d$ within which the spatial signature falls below the 50% of prevalence.

### Software

The data were described, analysed, and visualised in R 4.1.2 [50]. The maps were created with the ggmaps package in R and background Landsat-8 image courtesy of the U.S. Geological Survey.

## Results

### Spatial signatures from cross-sectional surveys

**Fig. 1:**
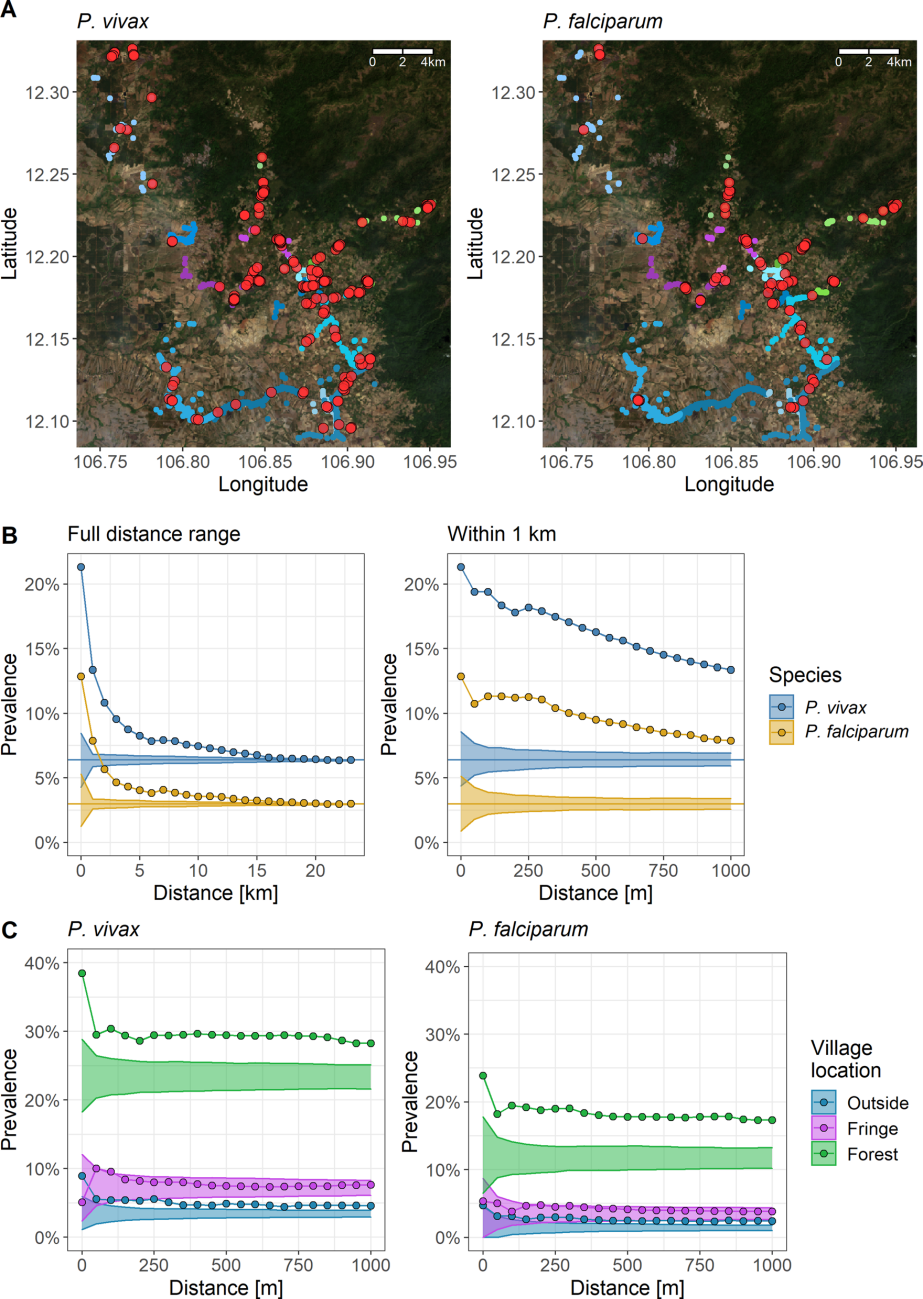
Spatial signature of *P. vivax* or *P. falciparum* infections in the Cambodian cross-sectional survey. Prevalence of infections at increasing distance around index infections. Panel **A**: Household locations in shades of blue, purple, or green per village outside the forest, at the forest-fringe, or inside the forest, respectively. *P. vivax* and *P. falciparum* infections in red. **B**: The spatial signature across the entire study region (left) or within 1 km around infections (right). Ribbon: 95%-quantile interval of null distribution. Horizontal line: Global study prevalence. **C**: Stratified by the villages’ forest proximity within 1 km

In the Cambodian cross-sectional survey, *P. vivax* and *P. falciparum* infections were found in all villages across the study region (Fig. 1A). Across all villages, the *P. vivax* prevalence was 6.4%, the *P. falciparum* prevalence was 3.0%, and the prevalence of co-infection was 1.1%. The prevalence of *P. vivax* around index infections decreased threefold from 21.3% at 0 km to the global study prevalence of 6.4% at maximum distance (Fig. 1B). The estimate at 0 km may include household members and inhabitants of the same building. For *P. falciparum*, prevalence decreased from 12.9 to 3.0%. Within 1 km, it decreased to 13.3% for *P. vivax* infections and to 7.9% for *P. falciparum* infections. Amongst the villages inside the forest, *P. vivax* prevalence decreased from 38.4% at 0 km to 28.2% within 1 km (Fig. 1C). For *P. falciparum*, prevalence fell from 23.9 to 17.3%. For both species, prevalence remains spatially clustered beyond the null distribution within 1 km amongst the villages inside and outside the forest but to a lesser degree, if at all, amongst the villages at the forest fringe.

**Fig. 2:**
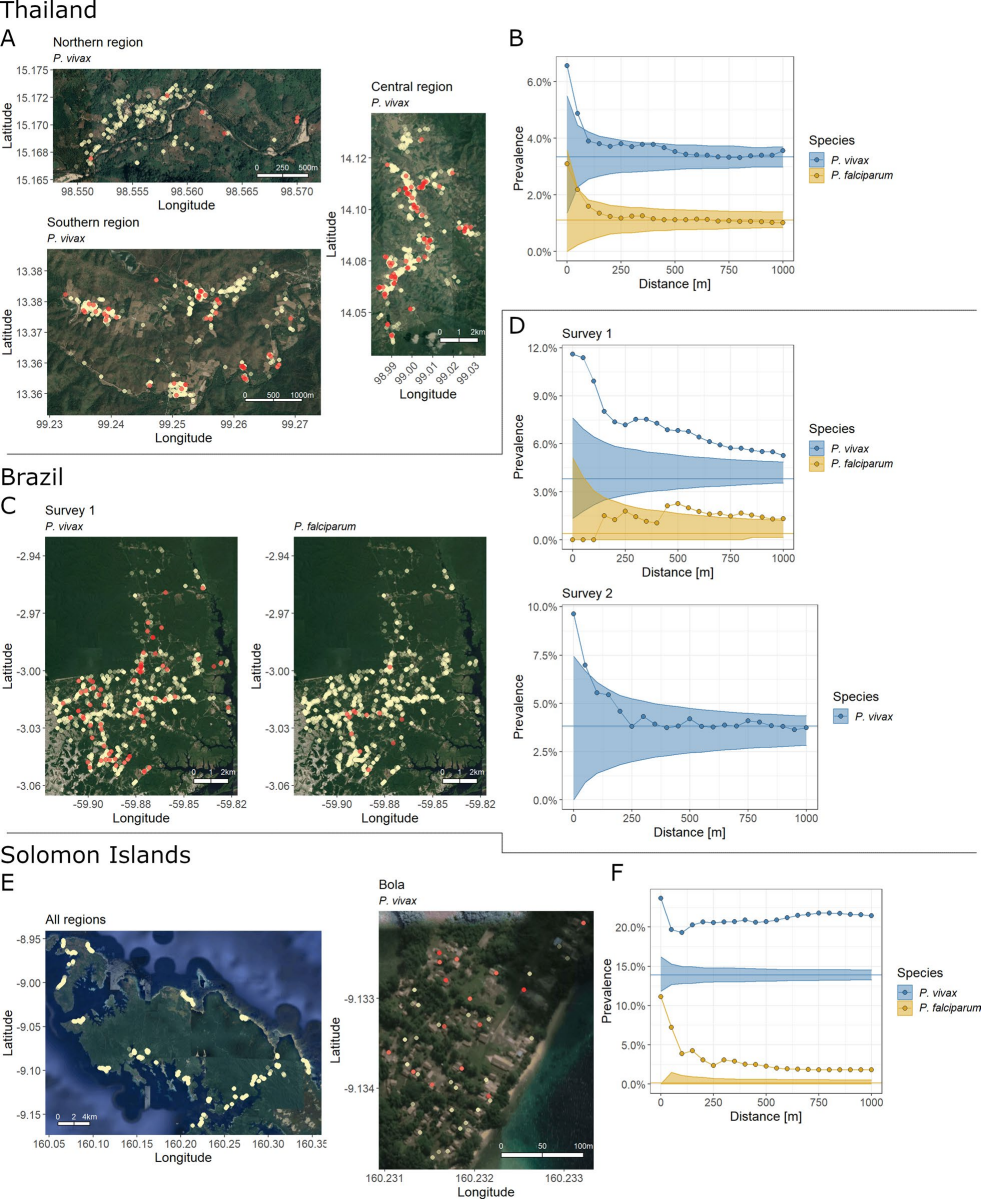
Spatial signatures of infections in the cross-sectional surveys in Thailand, Brazil, and Solomon Islands. Prevalence of *P. vivax* or *P. falciparum* infections at increasing distance around index infections in Thailand (upper panels **A**–**B**), Brazil (middle, **C**–**D**), and Solomon Islands (lower, **E**–**F**). Panels **A**, **C**, **E**: Maps with household locations in light gold. *P. vivax* (and for first Brazilian survey also *P. falciparum*) infections in red. Maps of *P. falciparum* infections in the Thailand survey, maps of the infections in the second Brazilian survey, and complete maps of *P. vivax* and *P. falciparum* infections in all surveyed villages in Solomon Islands are in the Supplementary Material. **B**, **D**, **F**: The spatial signatures per survey within 1 km around infections. Ribbon: 95%-quantile interval of null distribution. Horizontal line: Global study prevalence

In the cross-sectional survey across three sites in Western Thailand (Fig. 2A), prevalence of *P. vivax* infections around index infections decreased from 6.6% at 0 km to 3.6% at 1 km and for *P. falciparum* from 3.1 to 1.0% (Fig. 2B). Significant spatial clustering for *P. vivax* infections was found only within a radius of 75 m.

In the peri-urban part of Manaus, *P. vivax* and *P. falciparum* infections were detected in the first of the two cross-sectional surveys (Fig. 2C) and *P. vivax* infections in the second survey (maps in the Additional file 1: Figure S3). Only one *P. falciparum* infection was found in the second survey, precluding any signature analysis. *P. vivax* prevalence dropped from 11.4% at 0 km around index infections to 5.3% within 1 km in the first survey and from 9.6 to 3.7% in the second survey (Fig. 2D). Prevalence around *P. vivax* index infections was significantly elevated around index cases within 1 km in the first survey and up to 70 m in the second survey. Prevalence of *P. falciparum* infections in the first survey did not follow a decreasing pattern.

In the cross-sectional survey in Solomon Islands, *P. vivax* prevalence was 23.7% at 0 km around index infections and hovered between 19.3 and 21.8% until a distance of 1 km (a decreasing pattern is more apparent from the signature across the entire study region in the Additional file 1: Figure S5). For *P. falciparum*, prevalence decreased from 11.1% at 0 km around index infections until 1.8% within 1 km. Prevalence was elevated from the null distribution for both species across the distance of 1 km.

### Spatial and spatiotemporal signatures from cohort studies

**Fig. 3:**
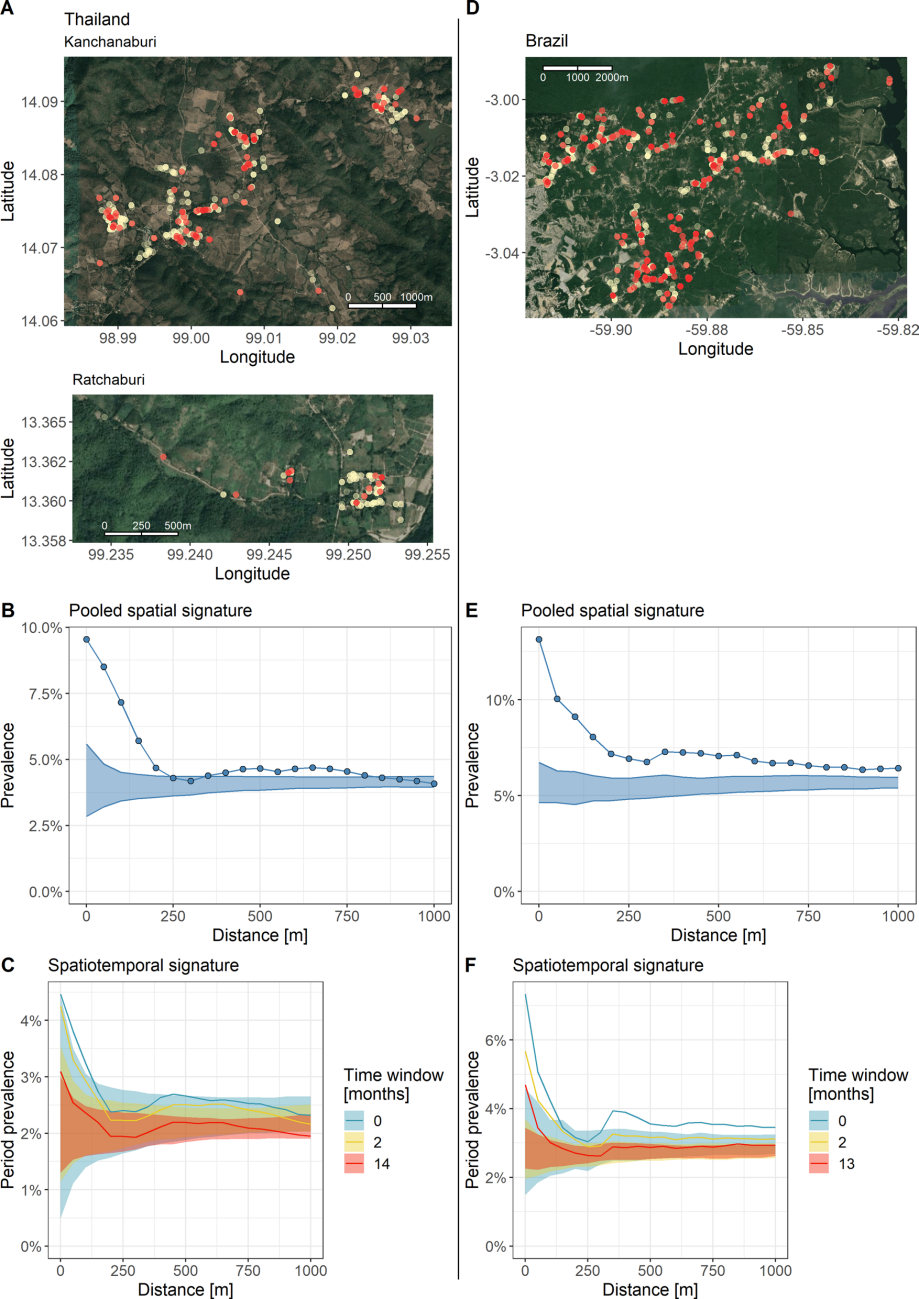
Spatial and spatiotemporal signatures of infections in the cohort studies in Thailand and Brazil. Prevalence of *P. vivax* infections at increasing distance (and in widening time windows) around index infections in Thailand (left, panels **A**–**C**) and Brazil (right, **D**–**F**). Panels **A**, **D**: Maps with household locations in light gold. *P. vivax* infections in red. **B**, **E**: The spatial signatures within 1 km around infections, pooling spatial clustering across the study visits. Ribbon: 95%-quantile interval of null distribution. **C**, **F**: The spatiotemporal signatures of incident *P. vivax* infections within 1 km and widening time windows in months

Pooling across the study visits of the longitudinal cohort studies, *P. vivax* prevalence around index infections per study visit decreased from 9.5% at 0 km to 4.1% at 1 km in the study in Thailand (Fig. 3B) and from 13.1 to 6.4% in the Brazilian study (Fig. 3E). The signature remains above the null distribution until 250 m in the Thai study and entirely within 1 km in the study in Brazil.

In the cohort study in Thailand, *P. vivax* period prevalence decreased from 4.5% at 0 km around incident infections to 2.3% within 1 km and the same study month, from 4.3 to 2.3% within 2 months, and from 3.1 to 1.9% across the entire study period of 14 months (Fig. 3C). Period prevalence was above the null distribution until 150 m within the same study month and within 2 months, and 25 m for infections across the study period.

In the Brazilian cohort study, *P. vivax* period prevalence fell from 7.3% at 0 km to 3.5% within 1 km and the same study month, from 5.7 to 3.1% within 2 months, and from 4.7 to 2.9% across the entire study period of 13 months (Fig. 3F). Period prevalence was above the null distribution until 150 m within the same study month, until 250 m for infections within 2 months, and 100 m for infections across the study period.

**Fig. 4:**
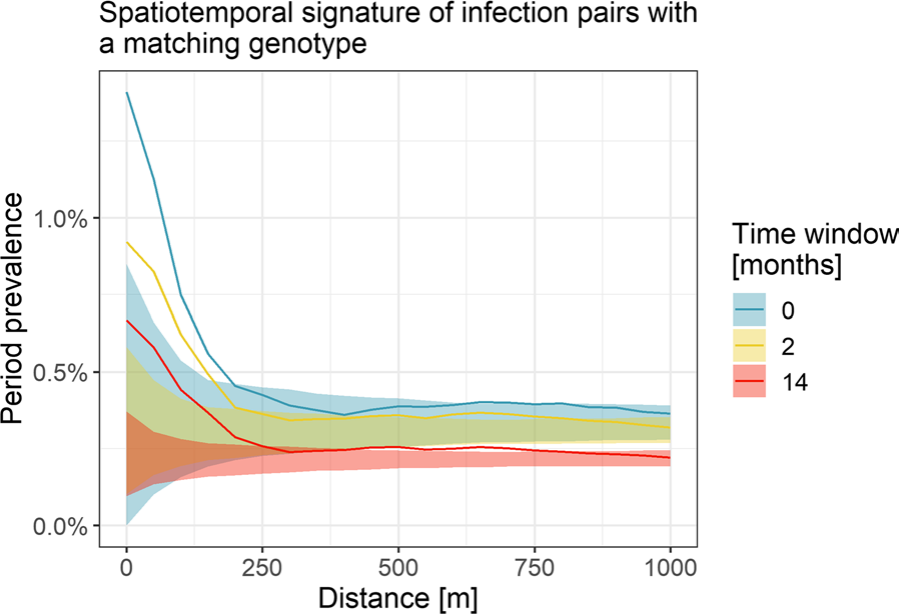
Spatiotemporal signatures of infections with a matching genotype in the cohort study in Thailand. Period prevalence of *P. vivax* infections at increasing distance (and in widening time windows) around index infections. Ribbon: 95%-quantile interval of null distribution

Considering only infection pairs with a matching genotype in the cohort study from Thailand, *P. vivax* period prevalence decreased from 1.4% at 0 km to 0.4% within 1 km and the same study month, from 0.9 to 0.3% for infections up to 2 months apart, and from 0.7 to 0.2% across the entire study period of 14 months (Fig. 4). Period prevalence was above the null distribution until 200 m within the same study month, until 250 m for infections within 2 months, and 250 m for infections across the study period.

### Stronger spatial clustering at lower global prevalence

**Fig. 5:**
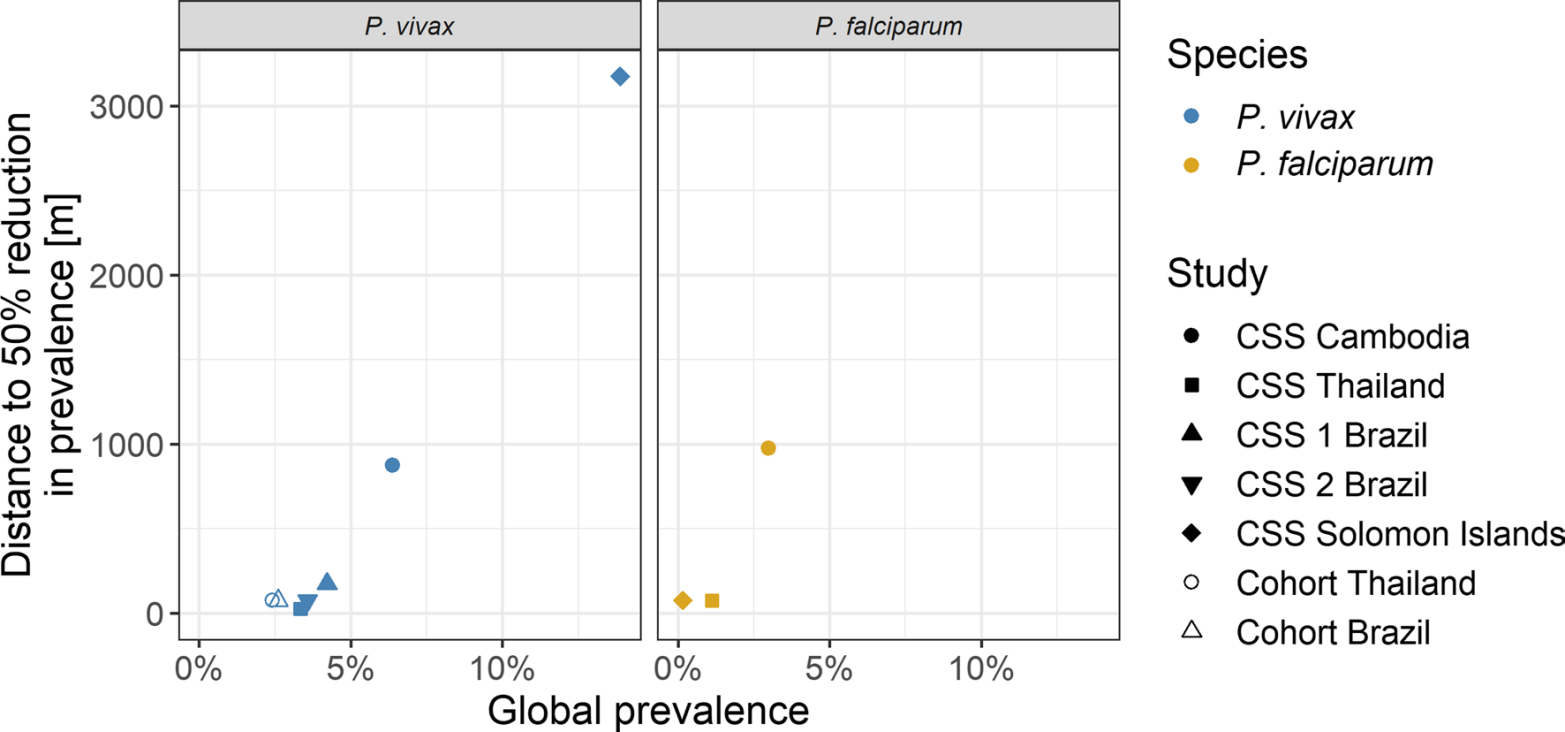
Comparative analysis of distance to 50% reduction in prevalence by global study prevalence. The distance within which the prevalence around infections has fallen by 50%, across the spatial signatures of the cross-sectional surveys and cohort studies. *CSS* cross-sectional survey

Across the spatial signatures of the cross-sectional surveys and cohort studies, the distance within which prevalence is reduced by 50% varies from 3175 m in the Solomon Islands survey to 25 m in the Thailand survey for *P. vivax* and from 975 m in the Cambodian survey to 75 m in the Thailand and Solomon Islands surveys for *P. falciparum* (Table 2, Fig. 5). Overall, the distance to 50% reduction tends to be shorter with lower prevalence.

**Table 2:** Distance to 50% reduction in prevalence and global study prevalence across the studies

Study type	Study site	*P. falciparum*		*P. vivax*	
		Global study prevalence (%)	Distance to 50% reduction in prevalence	Global study prevalence (%)	Distance to 50% reduction in prevalence
Cross-sectional surveys	Cambodia	3.0	975 m	6.4	875 m
	Thailand	1.1	75 m	3.3	25 m
	Brazil Survey 1	–	–	4.2	175 m
	Brazil Survey 2	–	–	3.6	75 m
	Solomon Islands	0.2	75 m	13.9	3175 m
Cohort studies	Thailand	–	–	2.4	75 m
	Brazil	–	–	2.6	75 m

From the spatial signatures (for cross-sectional surveys) or spatiotemporal signatures at time window of ‘0 months’, i.e. the same month (for cohort studies) for *P. vivax* and* P. falciparum*

## Discussion

Across multiple studies in diverse malaria-endemic regions, clustering of *P. vivax* and *P. falciparum* infections in close proximity around index infections was demonstrated, decreasing with distance. Similarly, co-infections with both species cluster spatially (Additional file 1: Figure S6). Comparing the signature to a null distribution after random re-allocation of infection locations shows statistically significant clustering in most settings. In a village in Senegal, spatial clustering of *P. falciparum* infections has previously been shown with this method [14]. The finding of spatial/spatiotemporal clustering is in line with previous studies close to the study sites [23, 24, 40, 41] or in other malaria-endemic regions [22, 25–28, 30–39, 42–44], deploying the SaTScan scanning method.

Clustering for *P. falciparum* was not found in the first Brazilian cross-sectional survey. Given very low global *P. falciparum* prevalence in both surveys [6], this could be due to a lack of statistical power. Considering the distribution of infection locations on the map, almost all infections surround a forested, sparsely inhabited area in which no samples were collected. The resulting spatial signature is possibly an artefact due to a peculiar distribution of household locations in an insufficiently sampled transmission hotspot.

The signature of *P. falciparum* infections in the cross-sectional survey in Thailand also lacks statistical significance, however follows a decreasing form. This lack of significance most likely stems from low prevalence and insufficient statistical power. Clustering of *P. vivax* infections in this and the second Brazilian survey was significant, however only within a short distance. Statistical power decreases with fewer cases and a tendency of clustering towards shorter distances was observed at lower global survey prevalence. Clustering might thus become increasingly difficult to demonstrate with statistical significance as prevalence decreases.

A significant spatial signature was also not observed in the Cambodian cross-sectional survey among the villages at the forest fringe. This is most likely because exposure of inhabitants of villages outside the forest is mainly driven by forest-going behaviour [9], and vectors in this region are mainly found in the forests [12].

*Plasmodium vivax* infection distribution in Solomon Islands’ cross-sectional survey showed significant clustering in a flat signature within 1 km distance around index infections, with clustering decreasing at greater distances. There was substantial variation in malaria prevalence between villages [48]. By calculating a spatial signature pooled across all villages that were sampled in the survey, significant within-village clustering was potentially missed in high transmission areas.

Spatial clustering was also shown in the cohort studies, particularly within shorter time windows. The amplitude of the signatures flattens with increasing time windows, demonstrating temporal clustering. Relapses could affect the spatiotemporal signature for *P. vivax*. Repeat infections in the same individual do not contribute towards the presented spatiotemporal signatures as only the first PCR-positive sample was considered. Relapses may still cause a higher degree of measured spatiotemporal clustering of *P. vivax* infections in a geographic region as relapsing neighbours of an index case also contribute to the detected clustering. From a programmatic perspective, this would still make the signature informative to guide the detection of both new and relapse infections. Considering only the incident infection does not take into account that subsequent infections in the same person can also be new infections and may reduce the ability to detect correlations over time. More detailed genetic analysis would be required to delineate the direction of the effect [51].

Considering only infection pairs of a matching genotype, the *P. vivax* spatiotemporal signature in the Thai cohort becomes even more pronounced. While the genotyping methods used here lack the resolution to define separate transmission chains with certainty, the finding that infections with a matching genotype cluster in space and time corroborates the principle of the spatial signature method.

The study is subject to certain limitations. First, all included studies were based on household locations. In an area with peri-domestic vector exposure, spatial clustering and a decrease with distance based on household locations can be readily interpreted, mainly by the vectors’ host-seeking. The interpretation in areas with mainly behavioural exposure is less straightforward, e.g. in villages outside the forest in the Cambodian site [9]. Significant clustering of infections based on household location could still occur though, e.g. resulting from forest-goers tending to live closer to each other in the villages or more general clustering of household locations based on socio-economic factors. The fact that even in these settings clustering might be found is useful from a programmatic perspective.

Another limitation is that the form of the signatures depends on the locations of the households and villages that were sampled, i.e. the underlying distribution of pairwise distances in the data. Pooling across villages attempts to generalize the prevalence dynamics. Studies that sampled across adjacent villages (e.g. the Cambodian survey) are easily accessible for this approach. However, if sampled villages are far apart, it can complicate the interpretation (e.g. the survey in Solomon Islands). This limitation was controlled for by ensuring that no gaps occur in the pairwise distance distributions. However, the *P. falciparum* signature in the first Brazilian survey may be an example where these distributions cause artefacts in the signature.

It is generally acknowledged that malaria elimination efforts need to be spatially tailored towards areas where (the highest) transmission risk persists [10, 19]. The scanning method has proven effective for detecting and locating clusters of infections. However, the method detects a single or multiple hotspots, ordered by their likelihood. The localization of hotspots with the strongest clustering can distract from spatial clustering that also exists outside the statistically most significant cluster. While the assessment of village-level prevalence and scanning methods are very valuable for identifying local spatial structure (e.g. identifying hotspots), these tools provide limited information on the more general properties of the spatial nature of malaria infection. Also, while some studies found ‘stable hotspots’, i.e. recurring hotspots in the same area from 1 year to another [36–38], others did not find that temporal predictive value [32]. Repeating resource-intensive observational studies in order to regularly update hotspot mapping is not feasible for malaria elimination programmes.

Reactive interventions are part of the recommended toolbox for malaria elimination [10]. Unless they choose levels such as households or villages, malaria control programmes need to be informed on the radius around index infections in which to operate. Scanning methods report the spatial extent of the detected hotspots which is very specific to the investigated area and only partially generalizable. The spatial signature method allows to detect clustering, to quantify its magnitude, and shows its dynamics across increasing distance. For that purpose, it generalizes the spatial clustering across the entire study area and data points. From a programmatic perspective, it can inform a cost–benefit approach for the optimal selection of a radius around detected index infections, balancing the trade-off between the total number of households included, and the expected proportion of infections found. That clustering on the basis of household locations was found even in areas with occupationally driven exposure is encouraging. From a reactive intervention perspective, such socio-economic clustering may still allow effective targeting, regardless of the underlying dynamics.

The distance to 50% reduction in prevalence was considered a suitable measure for comparing the signatures across study sites. For both *P. vivax* and *P. falciparum*, a trend towards stronger spatial clustering was observed at lower global study prevalence. This suggests that reactive infection detection strategies could become increasingly effective in low transmission settings approaching malaria elimination.

## Conclusion

Spatial clustering of *P. vivax* and *P. falciparum* infections was demonstrated across a diverse set of study sites and transmission intensities. Introducing a novel method in spatiotemporal malaria epidemiology, the distance within which clustering occurs around index infections was quantified. These distances are often short, e.g. below 200 m, tending to lower values at lower global study prevalence. The spatial signature of *Plasmodium* spp. infections offers a new tool to extract insights on *Plasmodium* spp. epidemiology from observational epidemiological field studies. It also provides a method to inform reactive infection detection strategies regarding effective and feasible radius choices of interventions around detected infections.

## Supplementary Information

**Additional file 1: Figures S1–S5.** Description of data: Distributions of distance between pairs of study participants or between pairs of cases and all other study participants across the studies; maps of households and *P. falciparum* infections in the cross-sectional survey in Thailand; maps of households and *P. vivax* and *P. falciparum* infections in the second cross-sectional survey in Brazil; maps of households and *P. vivax* and *P. falciparum* infections in the villages of the cross-sectional survey in Solomon Islands; spatial signature of prevalence of *P. vivax* or *P. falciparum* infections in the cross-sectional survey in Solomon Islands across the full distance range. **Figure S6.** The spatial signature of prevalence of co-infections of *P. vivax* an *P. falciparum* infections in the cross-sectional survey in Cambodia across the full distance range (left) and within 1 km (right). Ribbon: 95%-quantile interval of null distribution. Horizontal line: Global survey prevalence.

## Data Availability

The observational studies from which data were used list their data availability statements in the original publications.

## Abbreviatons

CSS: Cross-sectional survey
GPS: Global positioning system
PCR: Polymerase chain reaction
