# Supplementary material for: The spatial signature of Plasmodium vivax and Plasmodium falciparum infections: quantifying the clustering of infections in cross-sectional surveys and cohort studies
Source: Malar J. 2023 Mar 4;22:75. doi: 10.1186/s12936-023-04515-4 (PMC9985228; doi:10.1186/s12936-023-04515-4)
Supplement: Supplementary file 1 — Additional file 1: Figures S1–S5. Description of data: Distributions of distance between pairs of study participants or between pairs of cases and all other study participants across the studies; maps of households and P. falciparum infections in the cross-sectional survey in Thailand; maps of households and P. vivax and P. falciparum infections in the second cross-sectional survey in Brazil; maps of households and P. vivax and P. falciparum infections in the villages of the cross-sectional survey in Solomon Islands; spatial signature of prevalence of P. vivax or P. falciparum infections in the cross-sectional survey in Solomon Islands across the full distance range. Figure S6. The spatial signature of prevalence of co-infections of P. vivax an P. falciparum infections in the cross-sectional survey in Cambodia across the full distance range (left) and within 1 km (right). Ribbon: 95%-quantile interval of null distribution. Horizontal line: Global survey prevalence. [file 12936_2023_4515_MOESM1_ESM.docx]

# Supplementary Material -

# The spatial signature of *Plasmodium vivax* and *P. falciparum* infections: Quantifying the clustering of infections in cross-sectional surveys and cohort studies

Mirco Sandfort, Wuelton Monteiro, Marcus Lacerda, Wang Nguitragool, Jetsumon Sattabongkot, Andreea Waltmann, Henrik Salje, Amélie Vantaux, Benoit Witkowski, Leanne J. Robinson, Ivo Mueller, Michael White


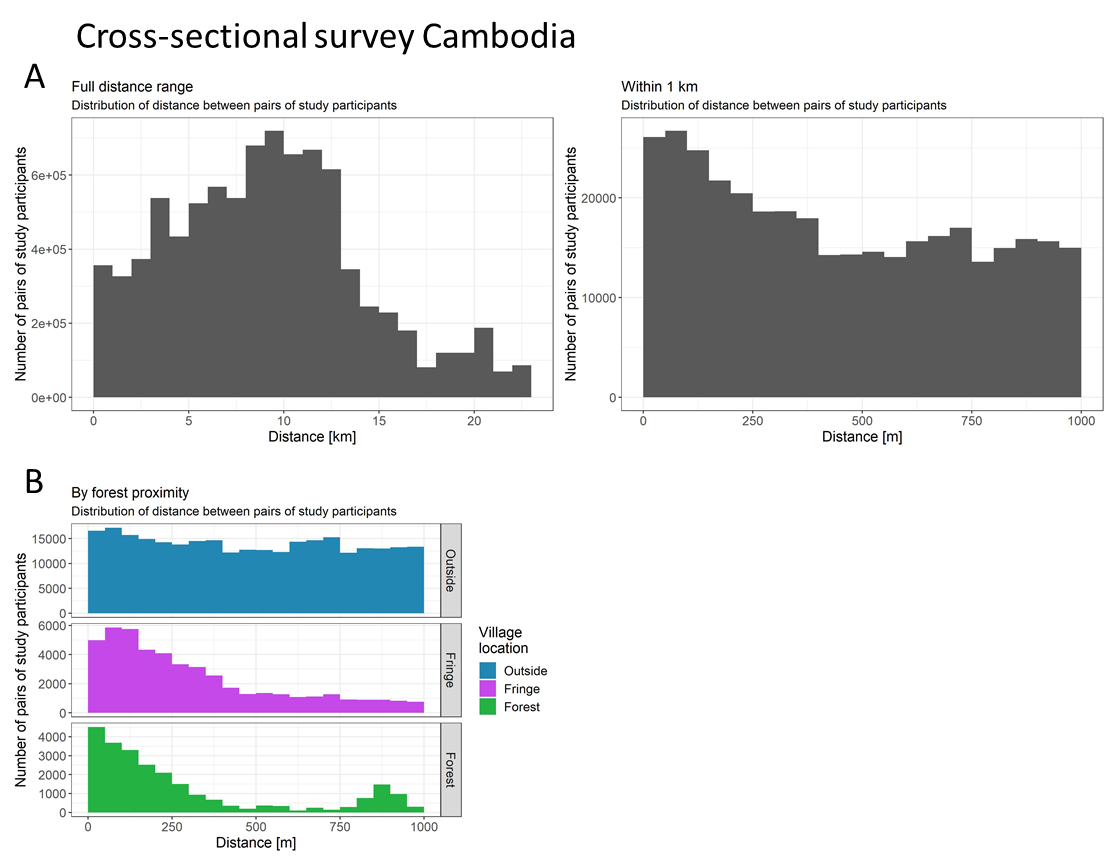


Fig. S1.1a Distribution of distance between pairs of study participants globally (panel A, left) and within 1 km (right) and stratified by the villages‘ proximity to the forest (panel B) for the cross-sectional survey in Cambodia.


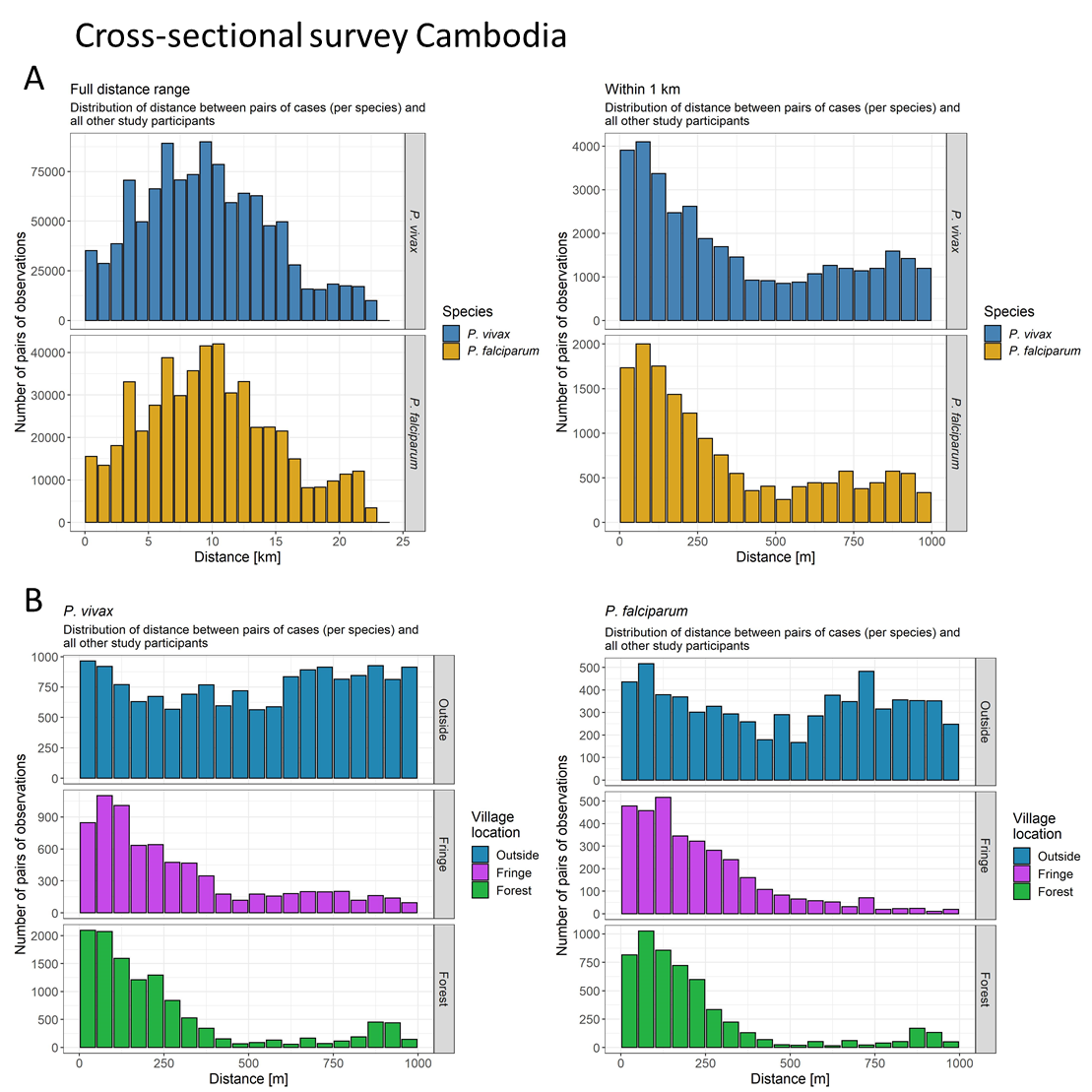


Fig. S1.1b Distribution of distance between pairs of cases and all other study participants globally (panel A, left) and within 1 km (right) per species for the cross-sectional survey in Cambodia. Stratified by the villages‘ proximity to the forest (panel B).


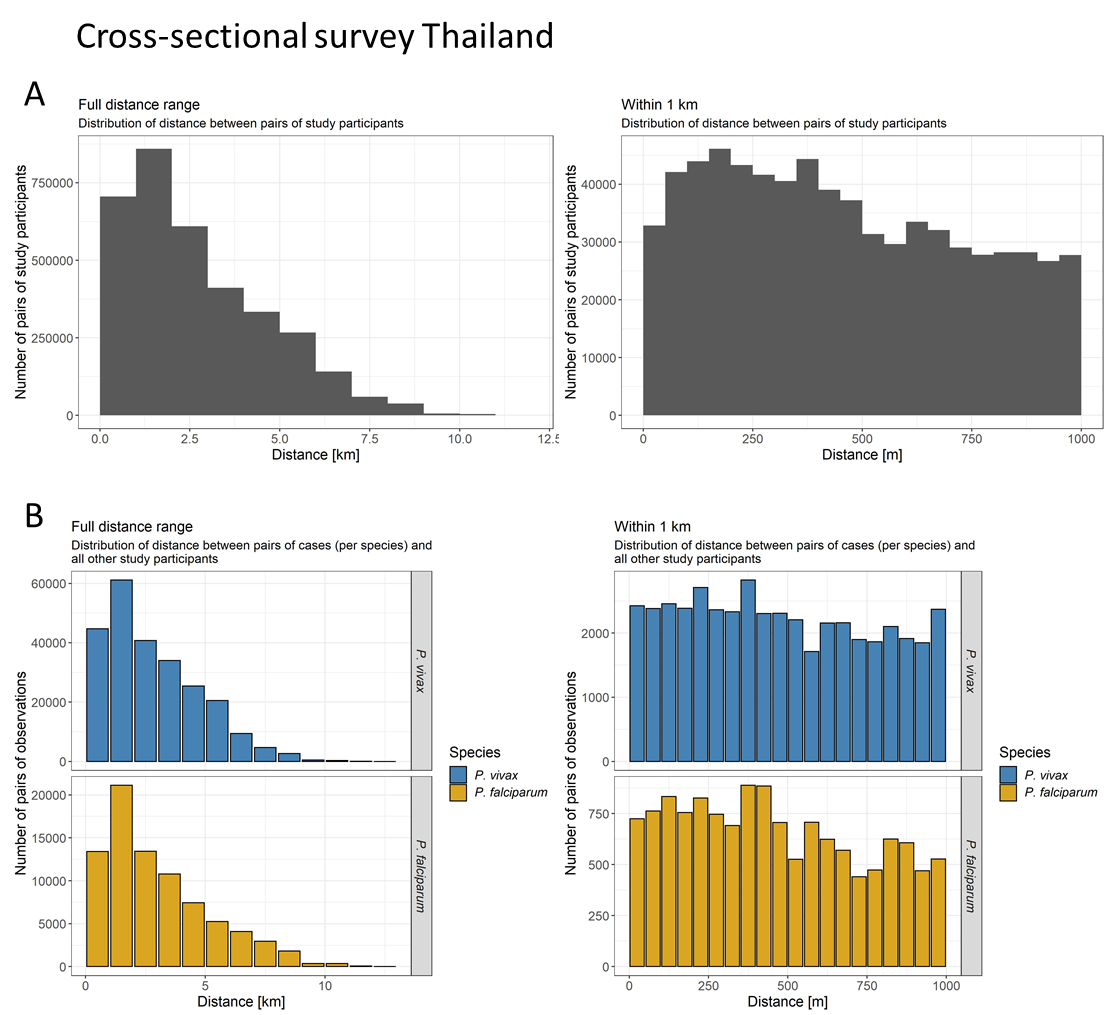


Fig. S1.2 Distribution of distance between pairs of study participants globally (panel A, left) and within 1 km (right) for the cross-sectional survey in Thailand. Distribution of distance between pairs of cases and all other study participants globally (panel B, left) and within 1 km (right) per species.


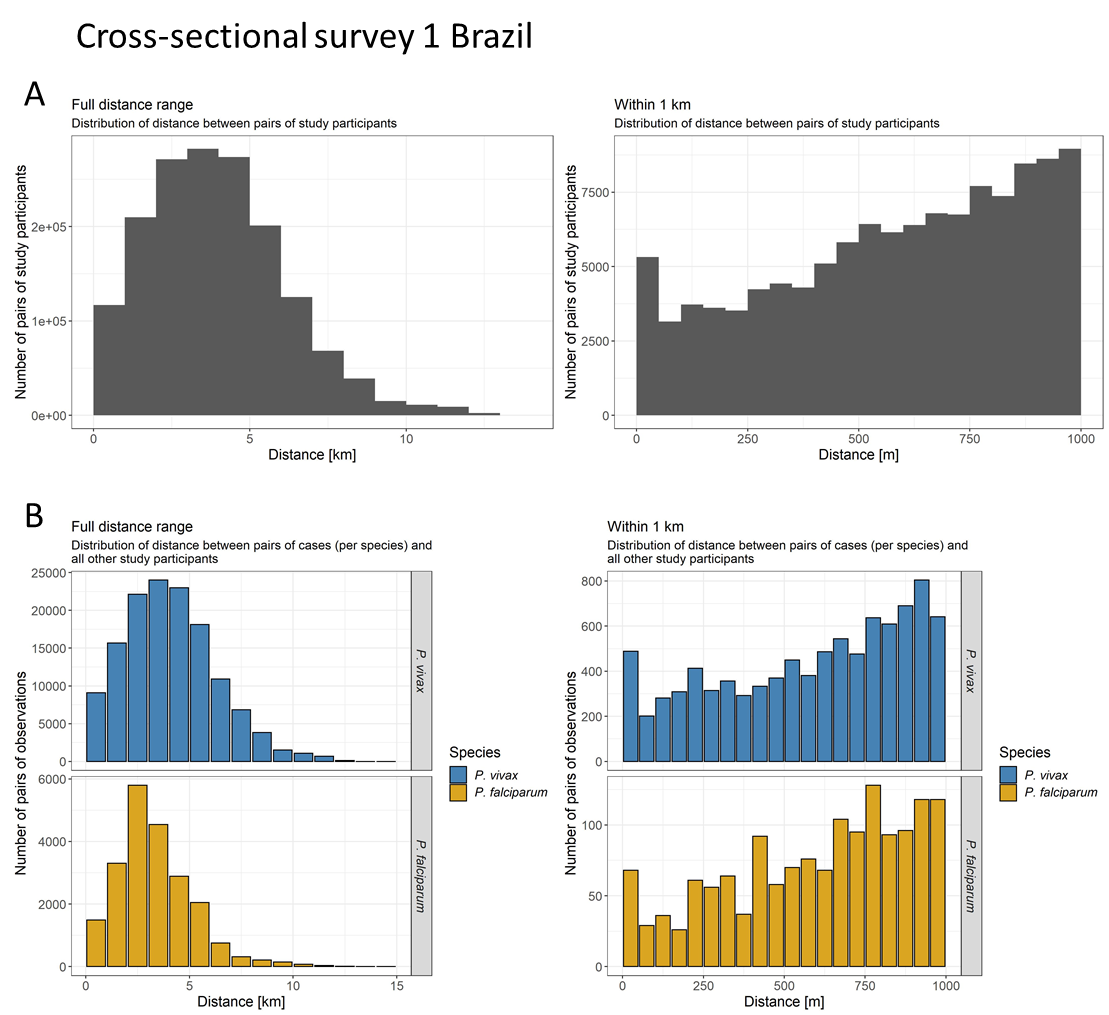


Fig. S1.3 Distribution of distance between pairs of study participants globally (panel A, left) and within 1 km (right) for the first cross-sectional survey in Brazil. Distribution of distance between pairs of cases and all other study participants globally (panel B, left) and within 1 km (right) per species.


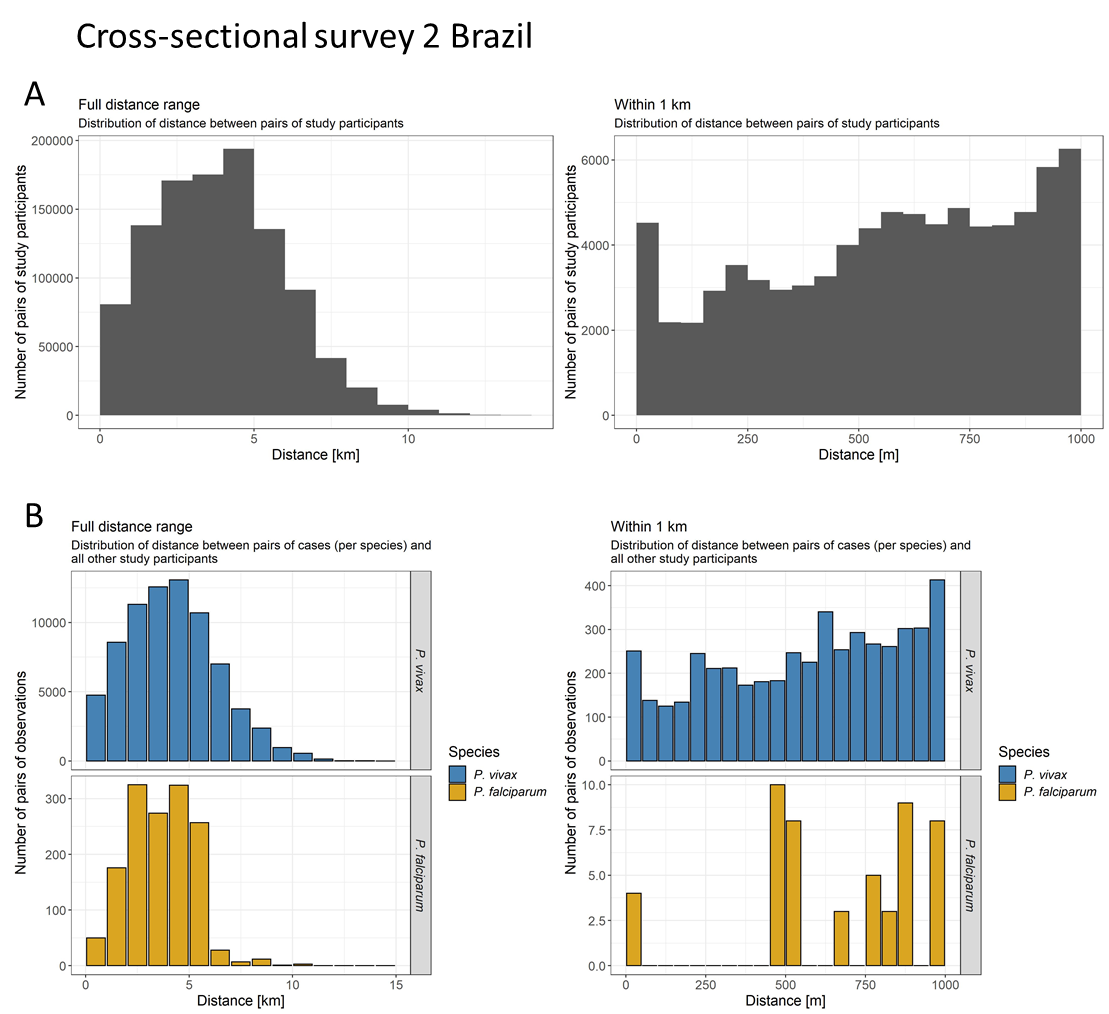


Fig. S1.4 Distribution of distance between pairs of study participants globally (panel A, left) and within 1 km (right) for the second cross-sectional survey in Brazil. Distribution of distance between pairs of cases and all other study participants globally (panel B, left) and within 1 km (right) per species.


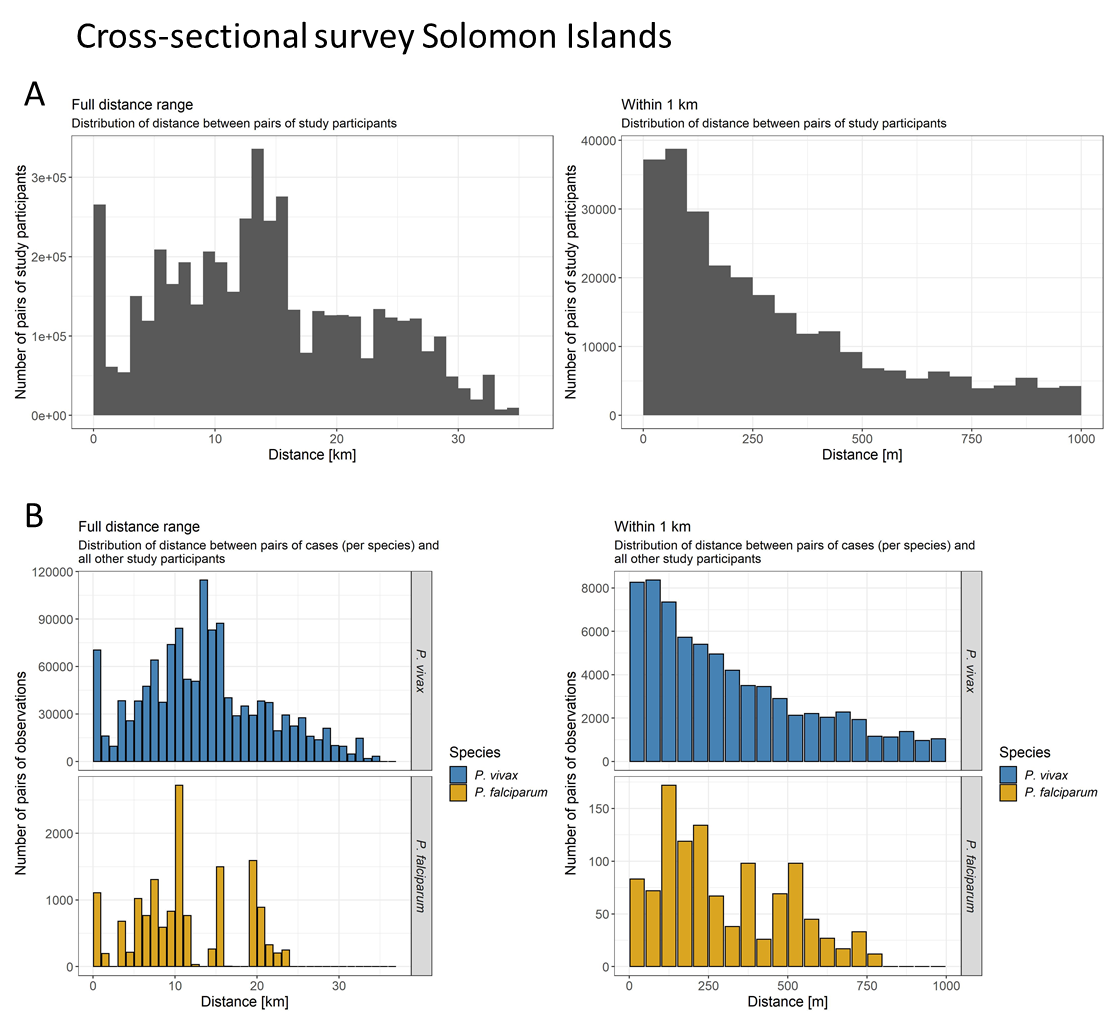


Fig. S1.5 Distribution of distance between pairs of study participants globally (panel A, left) and within 1 km (right) for the cross-sectional survey in Solomon Islands. Distribution of distance between pairs of cases and all other study participants globally (panel B, left) and within 1 km (right) per species.


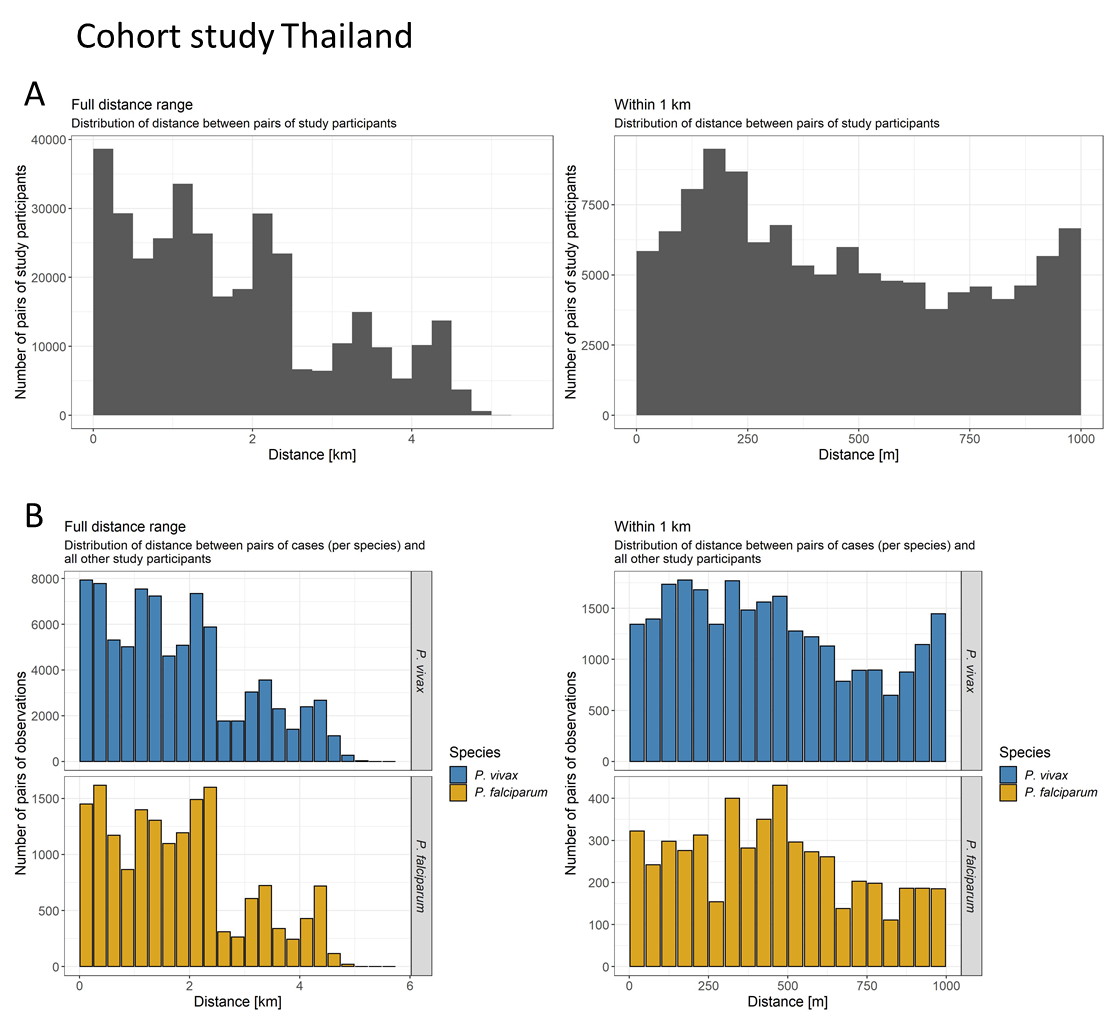


Fig. S1.6 Distribution of distance between pairs of study participants globally (panel A, left) and within 1 km (right) for the cohort study in Thailand. Distribution of distance between pairs of cases and all other study participants globally (panel B, left) and within 1 km (right) per species.


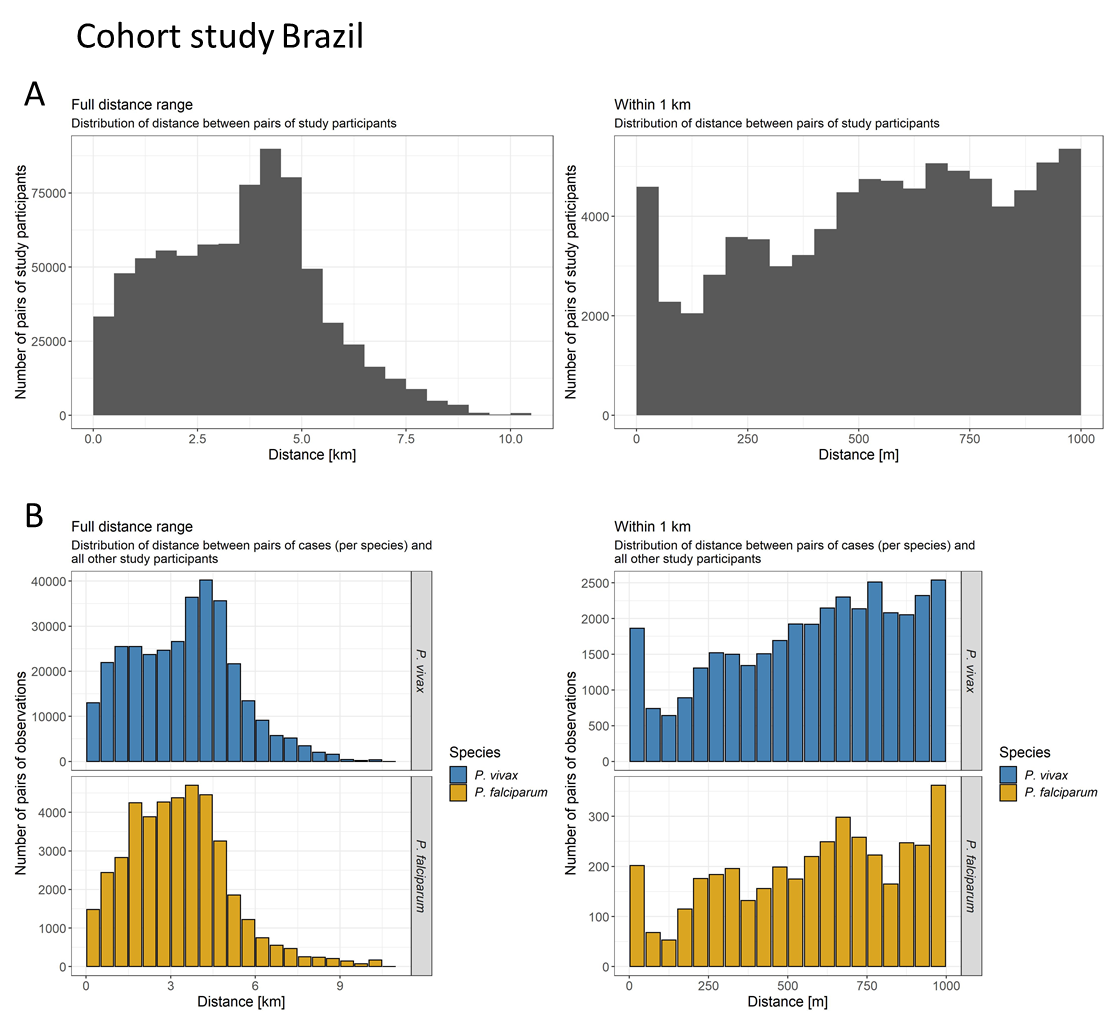


Fig. S1.7 Distribution of distance between pairs of study participants globally (panel A, left) and within 1 km (right) for the cohort study in Brazil. Distribution of distance between pairs of cases and all other study participants globally (panel B, left) and within 1 km (right) per species.


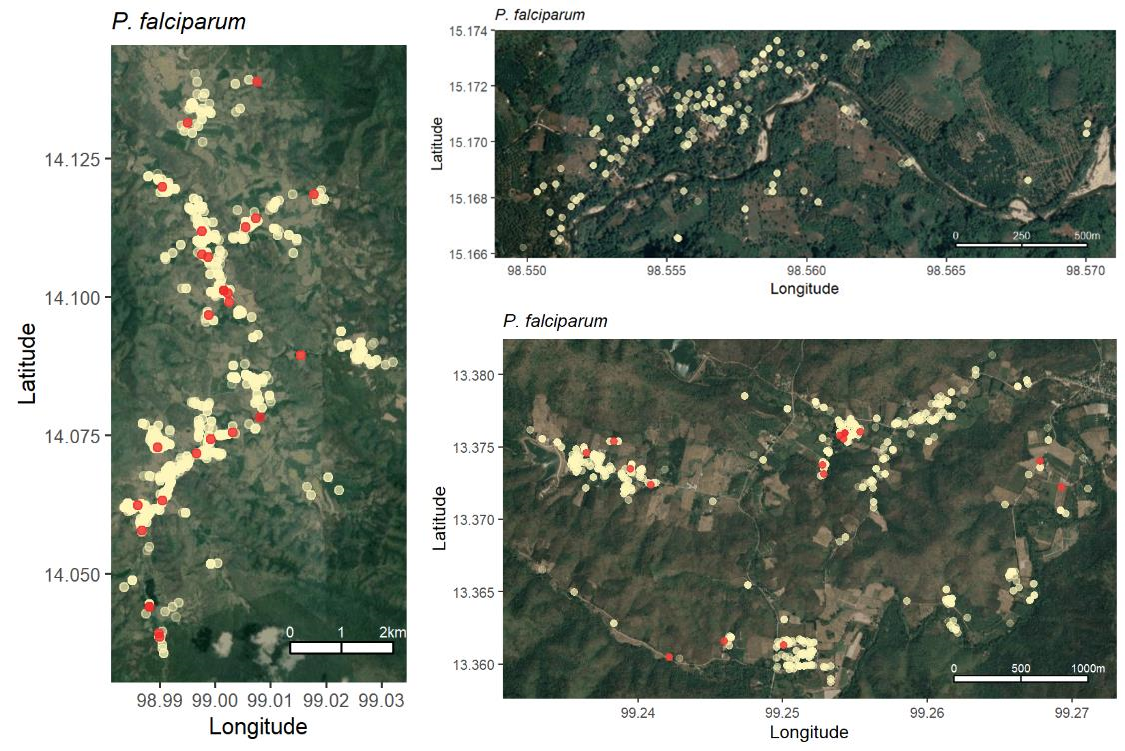


Fig. S2 Locations of households and *P. falciparum* infections in the cross-sectional survey in Thailand. Central region left panel, Northern region upper right panel, Southern region lower right panel. Household locations in light gold. Infections in red.


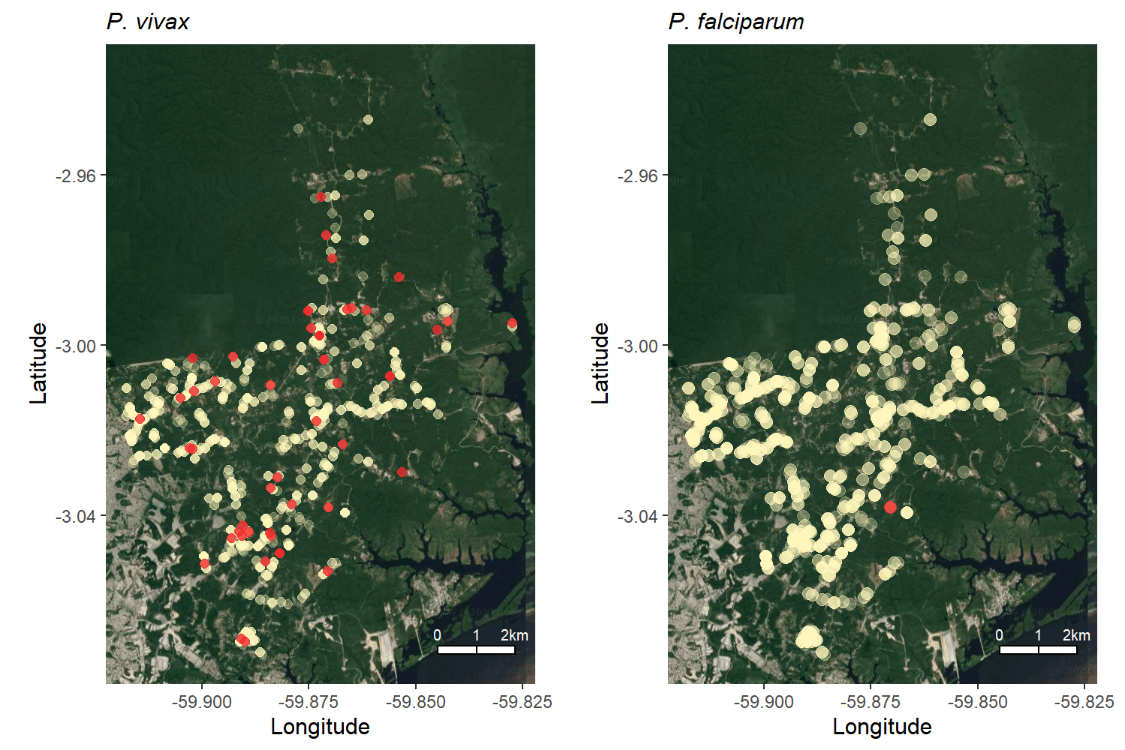


Fig. S3 Locations of households and *P. vivax* and *P. falciparum* infections in the second cross-sectional survey in Brazil. Household locations in light gold. Infections in red.


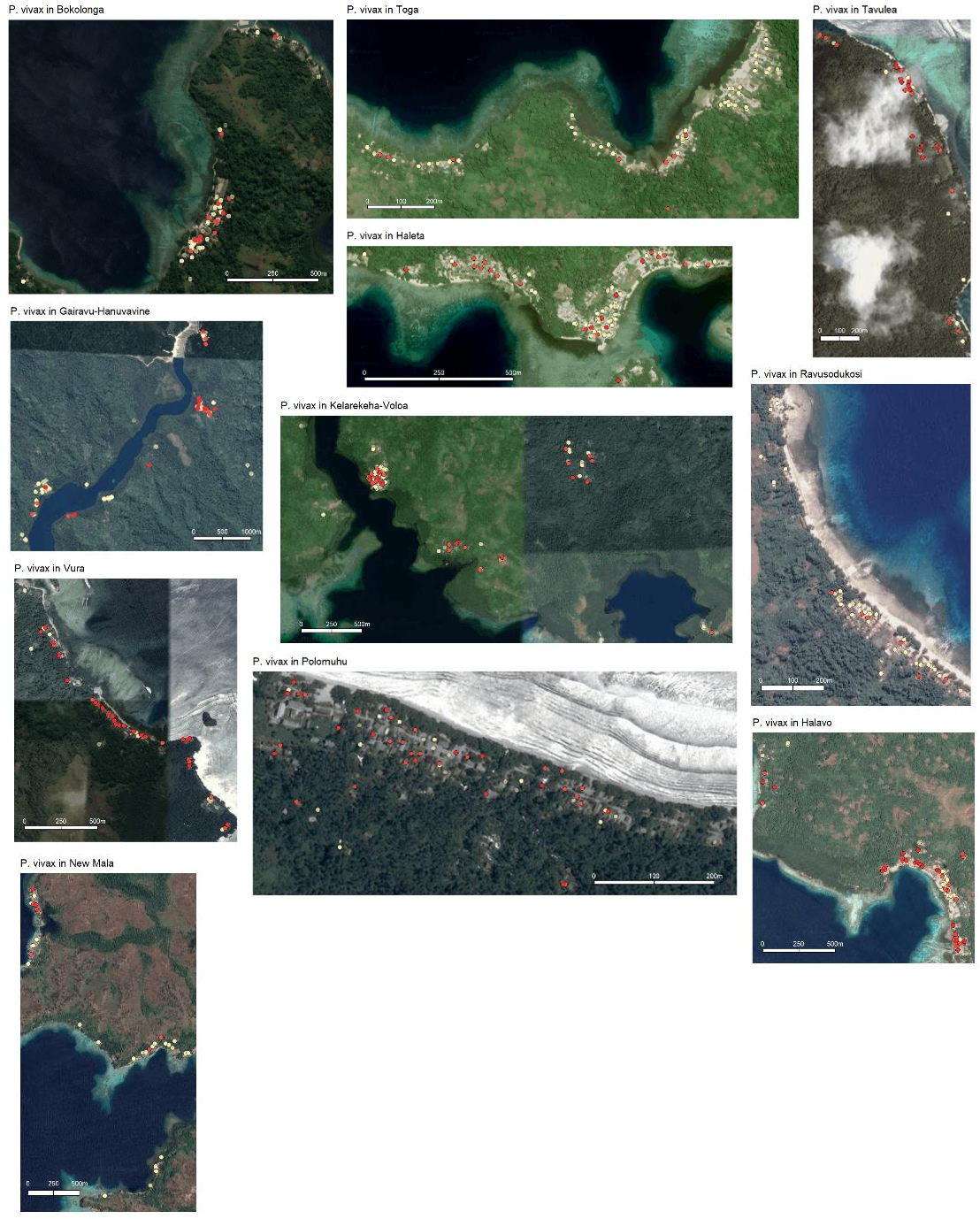


Fig. S4a Locations of households and *P. vivax* infections in the villages of the cross-sectional survey in Solomon Islands (not part of Figure 2 in the main text). Household locations in light gold. Infections in red.


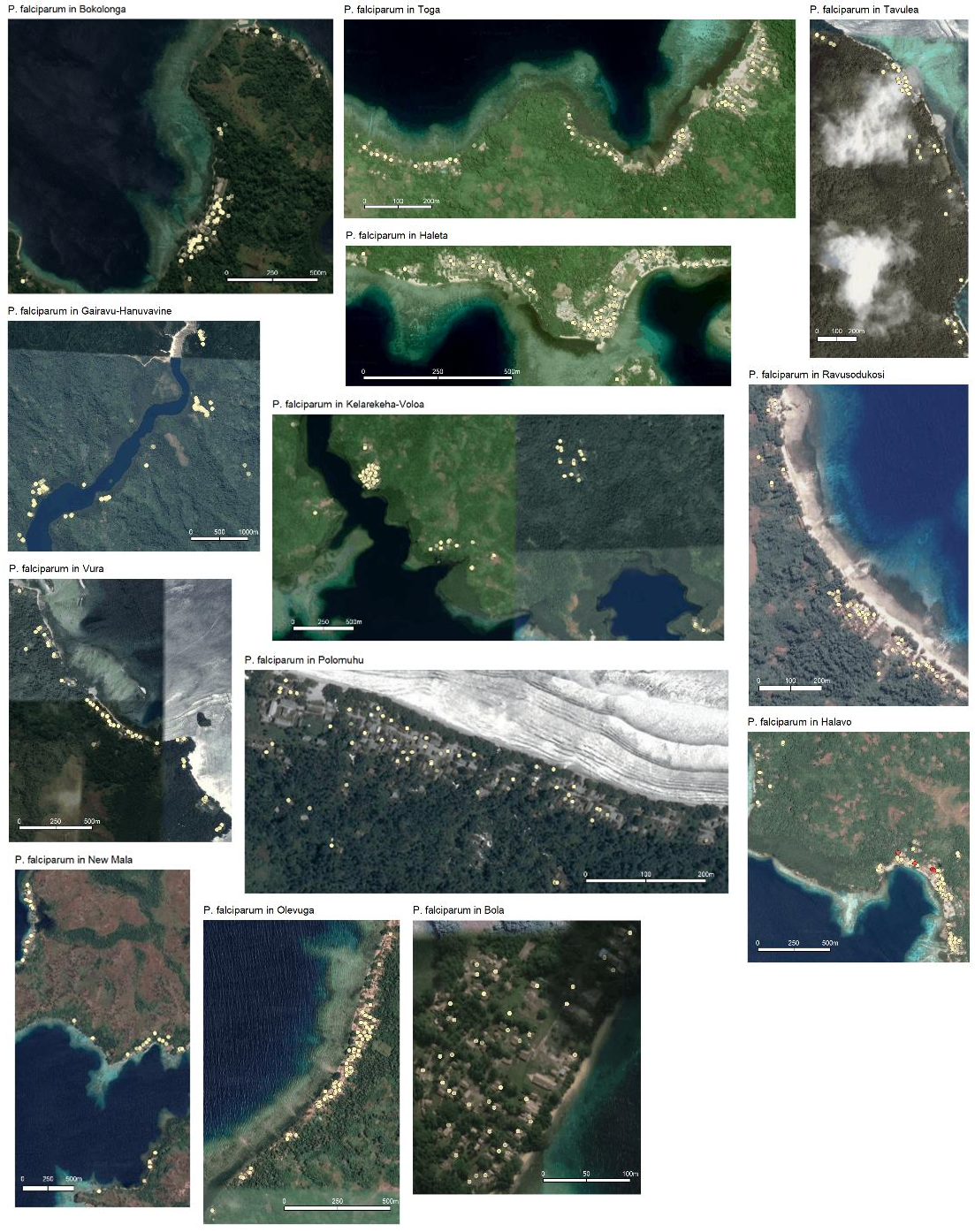


Fig. S4b Locations of households and *P. falciparum* infections in the villages of the cross-sectional survey in Solomon Islands. Household locations in light gold. Infections in red.


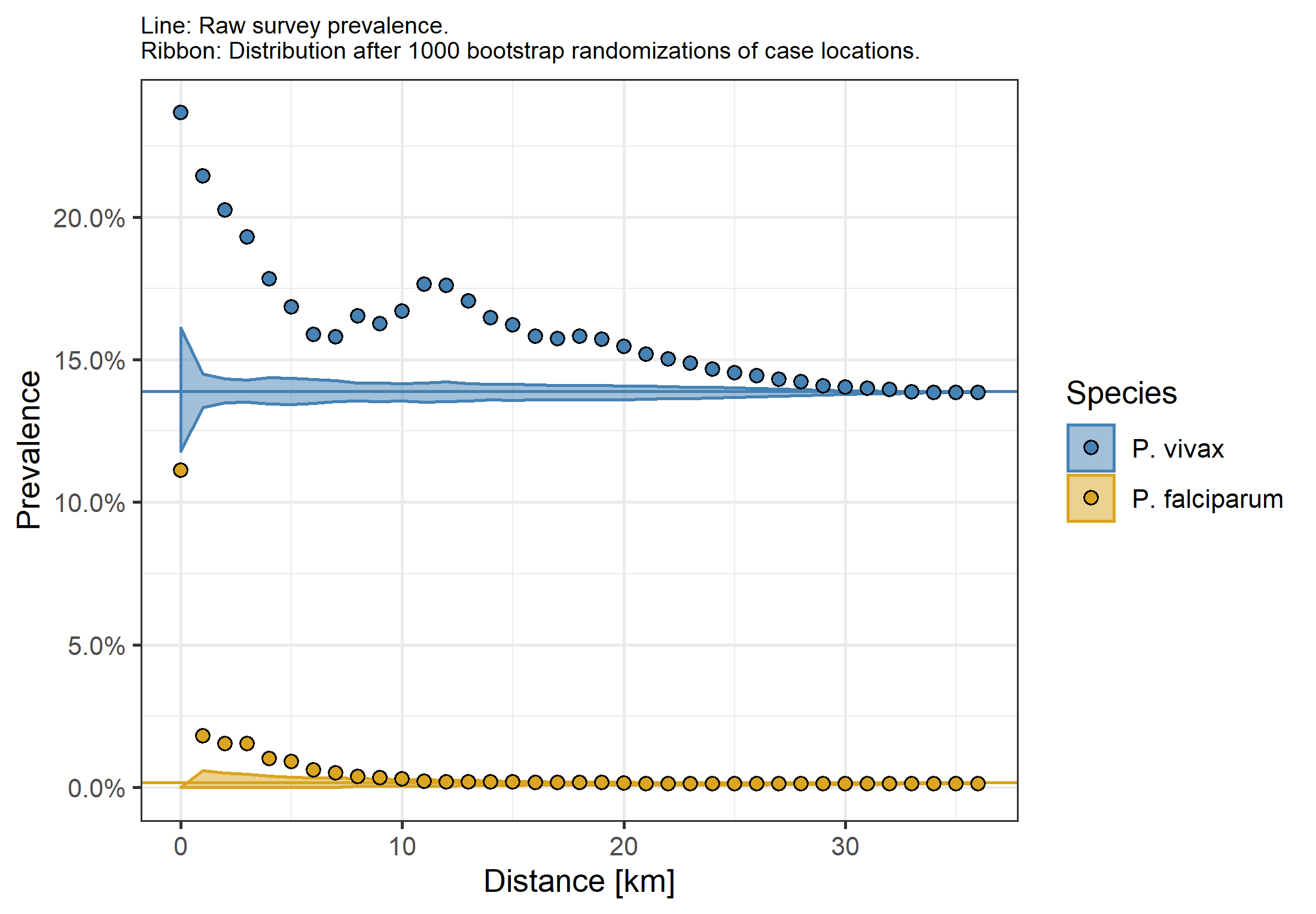


Fig. S5 The spatial signature of prevalence of *P. vivax* or *P. falciparum* infections in the cross-sectional survey in Solomon Islands across the full distance range. Ribbon: 95%-quantile interval of null distribution. Horizontal line: Global survey prevalence.


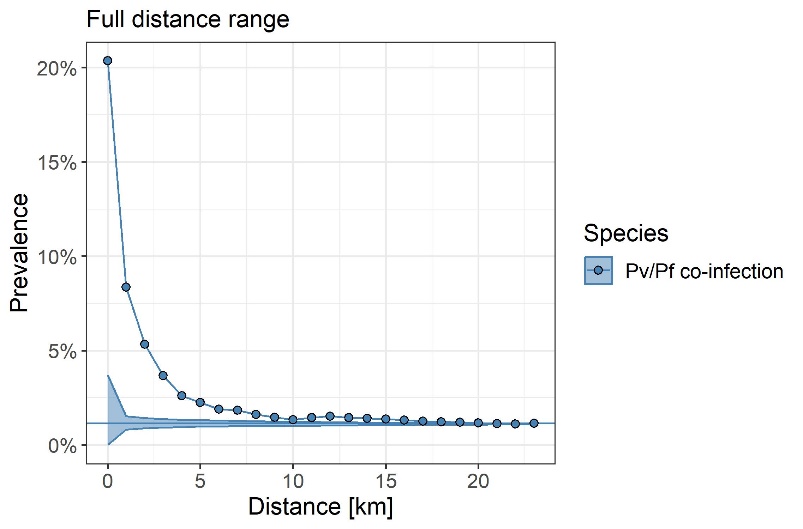

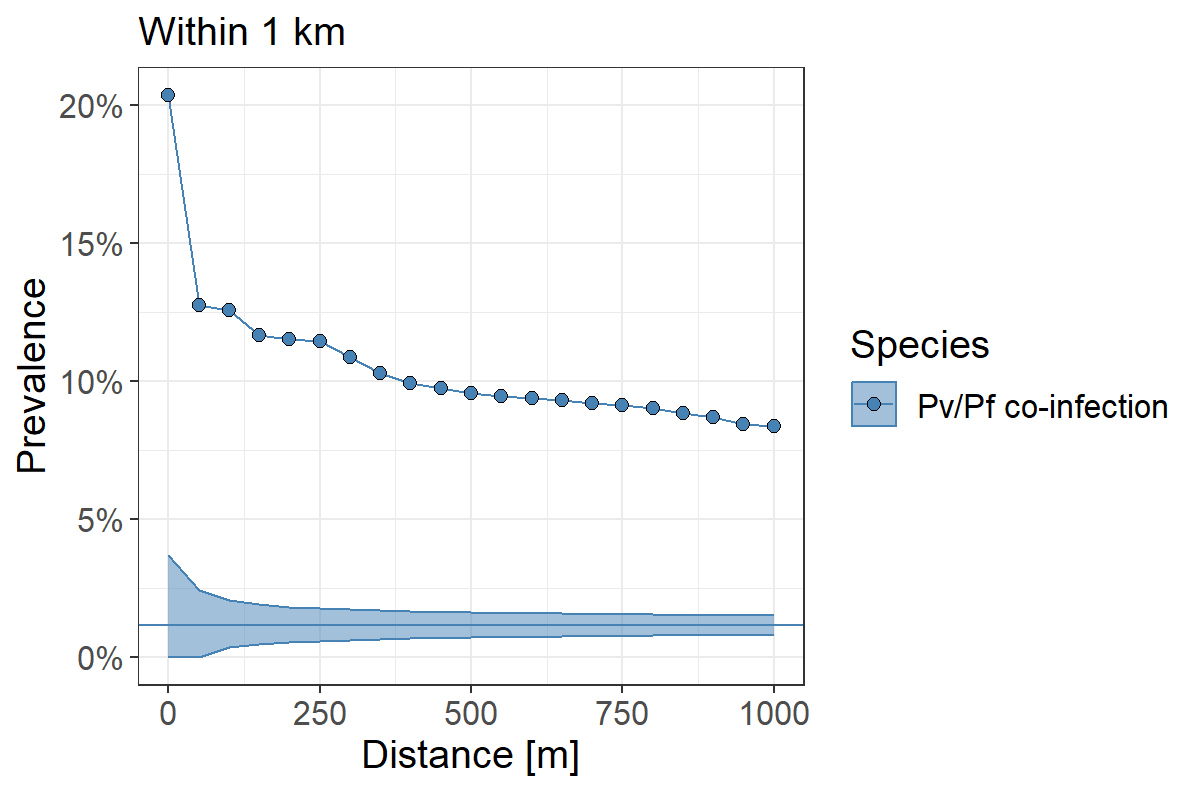


Fig. S6 The spatial signature of prevalence of co-infections of *P. vivax* an *P. falciparum* infections in the cross-sectional survey in Cambodia across the full distance range (left) and within 1 km (right). Ribbon: 95%-quantile interval of null distribution. Horizontal line: Global survey prevalence.
